# An open dataset for intelligent recognition and classification of abnormal condition in longwall mining

**DOI:** 10.1038/s41597-023-02322-9

**Published:** 2023-06-27

**Authors:** Wenjuan Yang, Xuhui Zhang, Bing Ma, Yanqun Wang, Yujia Wu, Jianxing Yan, Yongwei Liu, Chao Zhang, Jicheng Wan, Yue Wang, Mengyao Huang, Yuyang Li, Dian Zhao

**Affiliations:** 1 School of Mechanical Engineering, Xi’an University of Science and Technology, No.58, Mid-Yanta Road, Xi’an, 710054 China; 2 Shaanxi Key Laboratory of Mine Electromechanical Equipment Intelligent Detection and Control, No.58, Yanta Road, Xi’an, 710054 China; 3 MARCO automatic control system development Co.,LTD, No.20, Fenghui South Road, Xi’an, 710054 China

**Keywords:** Computer science, Energy access

## Abstract

The underground coal mine production of the fully mechanized mining face exists many problems, such as poor operating environment, high accident rate and so on. Recently, the intelligent autonomous coal mining is gradually replacing the traditional mining process. The artificial intelligence technology is an active research area and is expect to identify and warn the underground abnormal conditions for intelligent longwall mining. It is inseparable from the construction of datasets, but the downhole dataset is still blank at present. This work develops an image dataset of underground longwall mining face (DsLMF+), which consists of 138004 images with annotation 6 categories of mine personnel, hydraulic support guard plate, large coal, towline, miners’ behaviour and mine safety helmet. All the labels of dataset are publicly available in YOLO format and COCO format. The availability and accuracy of the datasets were reviewed by experts in coal mine field. The dataset is open access and aims to support further research and advancement of the intelligent identification and classification of abnormal conditions for underground mining.

## Background & Summary

Coal will remain the dominant energy source worldwide for decades to come^1^. Autonomous coal mining machines in longwall mining face can assist or replace human to complete the dangerous mining work, achieve safe and efficient production in coal mine. But it still needs human participation to complete some complex tasks. However, the underground coal excavation of fully mechanized longwall mining face exists many problems, such as poor operating environment, high disaster risk, high accident rate and so on. The intelligence mining has become one of the important ways to address the high-risk underground work, and achieve the goal of safe and efficient underground production^2^. With the rapid development of artificial intelligence technology, the abnormal situation of equipment, environment and personnel are expected to achieve real-time and accurate detection.

In a fully mechanized working face, hydraulic support is indispensable to the whole face’s safe production. As the core equipment for fully mechanized coal mining, hydraulic support can provide a safe working face, and to move the scraper conveyor and shearer in the working face^3^. It can also reliably and effectively support coal mine roof, isolate mined-out areas, prevent waste rock into the working face. In accordance with the coal mining process of the fully mechanized coal face, once the hydraulic support plate is not in place or not fully recovered during the working process, it may cause the movement interference between hydraulic support and shearer. Hence, it is necessary to find the status of the hydraulic support guard plate in time and deal with it accordingly. For the fully mechanized longwall mining face, large sized coal is easy to cause scraper conveyor blockage, retention and other abnormal state. It is necessary to automatically identify and track large coal, so as to timely judge and warn the abnormal state of large coal. Towline is used in fully mechanized mining face to ensure the power supply and stable operation of shearer. However, in the process of operation, the traction cable would be broken or be removed from the cable slot due to the stacking of cable clamps, and the cable may be torn off, resulting in underground electric leakage, which may eventually lead to electric shock, gas, coal dust explosion, fire and other major coal mine safety accidents. Therefore, it is necessary to conduct real-time status monitoring and intelligent analysis of the towline to ensure that the fault of the towline is detected and handled in time.

Aimed to protect the personnel safety of fully mechanized mining face, it is necessary to identify and track the mine personnel so as to judge whether the mine personnels are in a safe area. The personnel entering the dangerous area should be timely detected and positioned, the corresponding voice reminder processing should be carried out, and the operation of the corresponding equipment should be stopped at the same time. Except the mine workers entering dangerous areas, the coal miners will have a variety of different postures during work. In the complex working environment, the unsafe behaviours of miners will also easily lead to the increase of safety accidents in coal mine, and the abnormal behaviour of the downhole staff also needs attention at any time. Safety helmet is a kind of safety equipment that coal miners must wear at all times during their work. The area where the coal seam is extracted will cause the pressure to transfer from the hydraulic support to the coal wall, which may increase the pressure on the coal wall and eventually causes the phenomenon of coal wall spalling. The coal falling form roof and the collision between personnel and equipment may cause injury accidents. Hence, the safety helmets are related to the safety of coal miners in fully mechanized mining face, and the wearing of the safety helmet for the coal mine staff also needs real-time monitoring.

The above states of the hydraulic support guard plate, large coal, towline, mine worker detection, personal behaviour and the wearing condition of safety helmet are the key contents of abnormal detection and identification in fully mechanized longwall mining face. The monitoring video in fully mechanized mining face is numerous and updated quickly. The abnormal condition of the working face was judged by specialized personnel through real-time video surveillance in traditional production process, this may result in the abnormal condition not be found in time because the visual fatigue during the long-term work. Therefore, it is of great significance to apply artificial intelligence technology to the analysis, identification and warning of the abnormal state, which includes hydraulic support guard plate, large coal, towline, miners’ behaviour and the wearing condition of safety helmet. The object detection using intelligence data-mining is inseparable from datasets and a large number of samples are required for training to achieve better generalization^4–6^. Hence, it is very necessary to establish an image dataset to identify and warn the underground abnormal conditions of the fully mechanized longwall mining face. Considering that the downhole datasets are still blank at present, this work constructs image dataset DsLMF+ for intelligent recognition of abnormal condition in underground longwall mining face, which mainly consists of the hydraulic support guard plate, large coal, towline, mine safety helmet, coal miners and miners’ behaviour in the fully mechanized face.

Currently, datasets are widely used in automatic driving, object detection, face recognition, natural language processing, text detection, medical and other fields^7–10^. Some widely used object detection datasets are as follows: (1) COCO datasets with large-scale commonly used items as target detection objects^11–13^; (2) VOC datasets with people, common animals, traffic vehicles, indoor furniture objects as target detection objects^14–16^; (3) DOTA dataset with airplanes, ships, storage tanks, baseball stadiums, tennis courts, basketball courts, ground runways, ports, bridge as target detection objects^17–19^; (4) TT100K dataset with common vehicles as the target detection object^20–22^; (5) WIDER FACE dataset with facial expression, illumination and posture as target detection objects^23–25^; (6) YOLO format dataset that dedicated to the target detection^26–28^, etc. In addition to these common datasets, we can also customize the dataset through pytorch framework, but the custom dataset format is complex, diversified and poor sharing^29^. The downhole datasets are still blank at present, in order to construct and facilitate the promotion and application of image dataset of the fully mechanized face in the field of intelligent coal mining, the compatibility and practicability of the coal mine dataset should be taken into consideration.

On the basis of the analysis on the format and production method of the above commonly used object detection datasets, the production of the datasets in this work has been completed by personnels who are familiar with the fully mechanized mining face in coal mine. The Labelimg software has been used to complete the label annotation of datasets in the YOLO format^30^, which make it convenient to be used in the currently popular YOLO series target detection networks. At the same time, in order to extend the application range of this dataset, the label format of the dataset has also been converted into the COCO format through label format conversion script, and therefore it could be used in the currently popular COCO target detection methods. Of course, in addition to the COCO label format and the YOLO label format, the rest of data label format can also be converted through the tag conversion script.

The image dataset of the fully mechanized longwall mining face (DsLMF+) is of great significance for the application of object detection using intelligence data-mining in the field of coal mine, which is expected to be able to identify and warn the underground abnormal conditions, solving the problems of underground dangerous and inefficient work and thus accelerate the intellectualization of coal mine.

## Methods

**Fig. 1:**
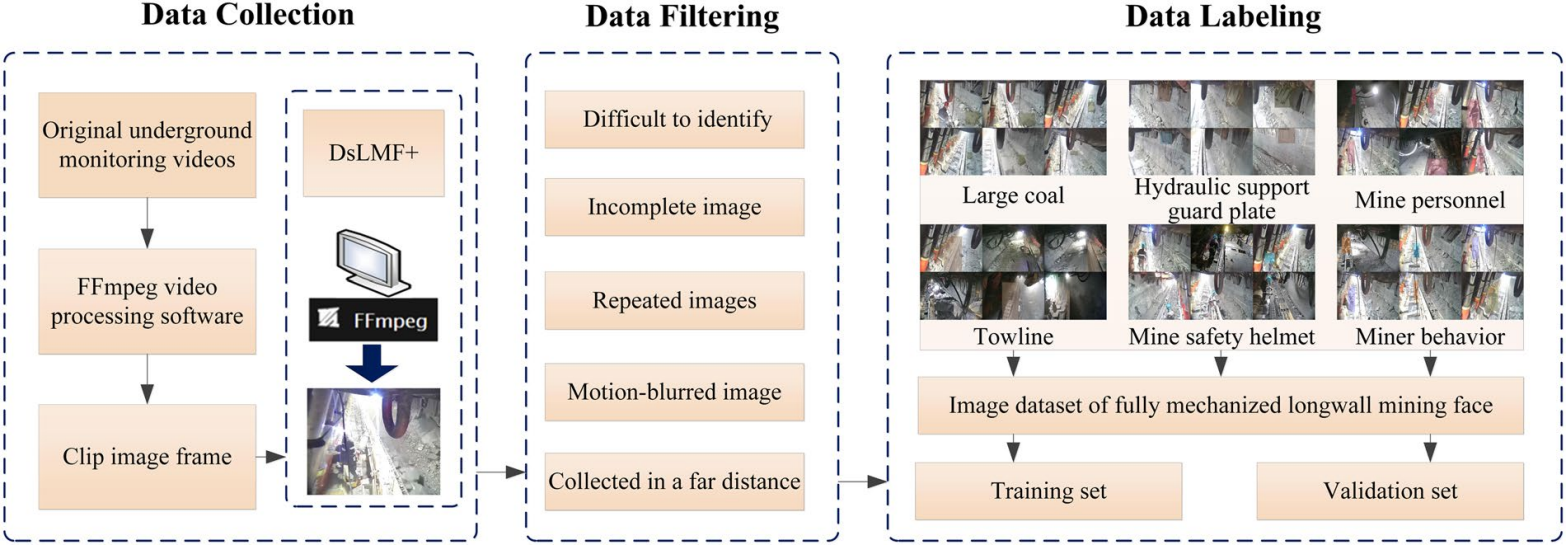
Overview of the construction process for the DsLMF+ datasets.

The construction process of the image dataset of underground longwall mining face (DsLMF+) is shown in Fig. 1, which is mainly divided into the following three steps: (1) Image data collection; (2) Image data filtering; (3) Data labeling.

### Image data collection

The original underground monitoring videos of the fully mechanized coal mining face were offered by several coal mines in Shaanxi Province of China, which were then screened and classified according to the different target object. We signed a scene authorization agreement with Shaanxi Coal and Chemical Industry Group Sunjiacha Longhua Mining Co.,LTD, so as to ensure that the dataset could be disclosed normally. Meanwhile, the agreement also included the authorization for the disclosure of the portraits of the mine personnel, so as to ensure that the miners who are photographed in the coal mine scene were aware of the disclosure of the dataset. The image acquisition equipment is composed of IVG-G5A network HD camera and Openmv IMX335(1/2.8”) lens. The lens focal length is 2.02 mm, and angle of field of view is 119.8°(D), 105.2°(H) and 87.2°(V). The camera can complete the image acquisition with a maximum resolution of 5 megapixels, the frame rate is 1~30FPS, and the used video formats are Flash video (FLV) and MPEG-4. The FFmpeg video processing software is used to process the needed classified videos^31^ and clip relevant images according to the different frame rate settings. The DsLMF+ datasets built in this work consists of 6 categories, which are respectively coal miners, large coal, towline, mine safety helmet, hydraulic support guard plate and miners’ behaviors. Considering that there is no target object to be annotated in some original images data, that is, the images do not include the mine personal, large coal, towline, hydraulic support guard plate and other target categories that need to be annotated. Therefore, some image frames have been removed and the other images are sorted according to the different categories, and the obtained images are used as the original image source of the DsLMF+ dataset.

### Image data filtering

The original image source of the DsLMF+ dataset will then be screened. The DsLMF+ dataset collected in this work mainly includes the mine personnel, large coal and hydraulic support guard plate, towline, mine safety helmet and miners’ behaviors, on account of that some images in original datasets might be with no target, incomplete target, and poor image quality that makes it difficult to identify the target, hence those images where might exist some abnormal data should be all removed.

The abnormal images that need to be processed mainly include the following situations: 1) When the fully mechanized mining face is affected by severe environmental factors such as high dust and water mist, it is difficult to identify the coal miners, large coal and hydraulic support guard plate, towline, mine safety helmet and miners’ behaviors in the collected images. 2) Due to the limited field of view of a camera or the occlusion, the target acquisition is incomplete in the process of image acquisition, resulting in only local features of the target are included in the acquired images. 3) When the fully mechanized mining face has stopped working, the camera still continues to collect images, resulting in a large number of repeated images in the collected video images. 4) The target objects in the downhole video acquisition are in a moving state. In the process of converting these videos into pictures, a reasonable frame rate should be adopted according to the different moving speed. However, if the target moves too fast, the picture obtained by video conversion will inevitably be blurred. 5) Due to the influence of the downhole environment and the distance between the target from the camera, the target object at a far distance is difficult to distinguish from other equipment.

All the above abnormal video images need to be manually or automatically eliminated in the process of image dataset production. In order to make it reproducibility of the datasets, we used ResNet50 to build a tri-classification automatic filtering network model to deal with the low-quality images that affected by downhole environmental factors such as high dust, water mist, motion blur, etc. In this work, some high dust and water mist images, defocused and motion blurred image as well as clear image were selected from the collected raw images data, and constructed an image filtering dataset for the training and verification of the tri-classification automatic filter model. The obtained automatic filter model can be used to deal with the invalid images data automatically to increase the reproducibility of our datasets and enhance the chances for other researchers to collaborate with the datasets. The tri-classification automatic filter model has been provided along with the datasets, and its specific usage can be on reference on in its attached README file. In addition, the structural similarity index SSIM can be used to judge and automatically filter out the duplicate or similarity images. For the other cases, considering that it is easy to be affected by personal subjective factors in the process of screening images, the multiple people uniformly reviewed the controversial images in the dataset when removing images from the dataset, especially for those images that are difficult to distinguish.

### Data labeling

**Fig. 2:**
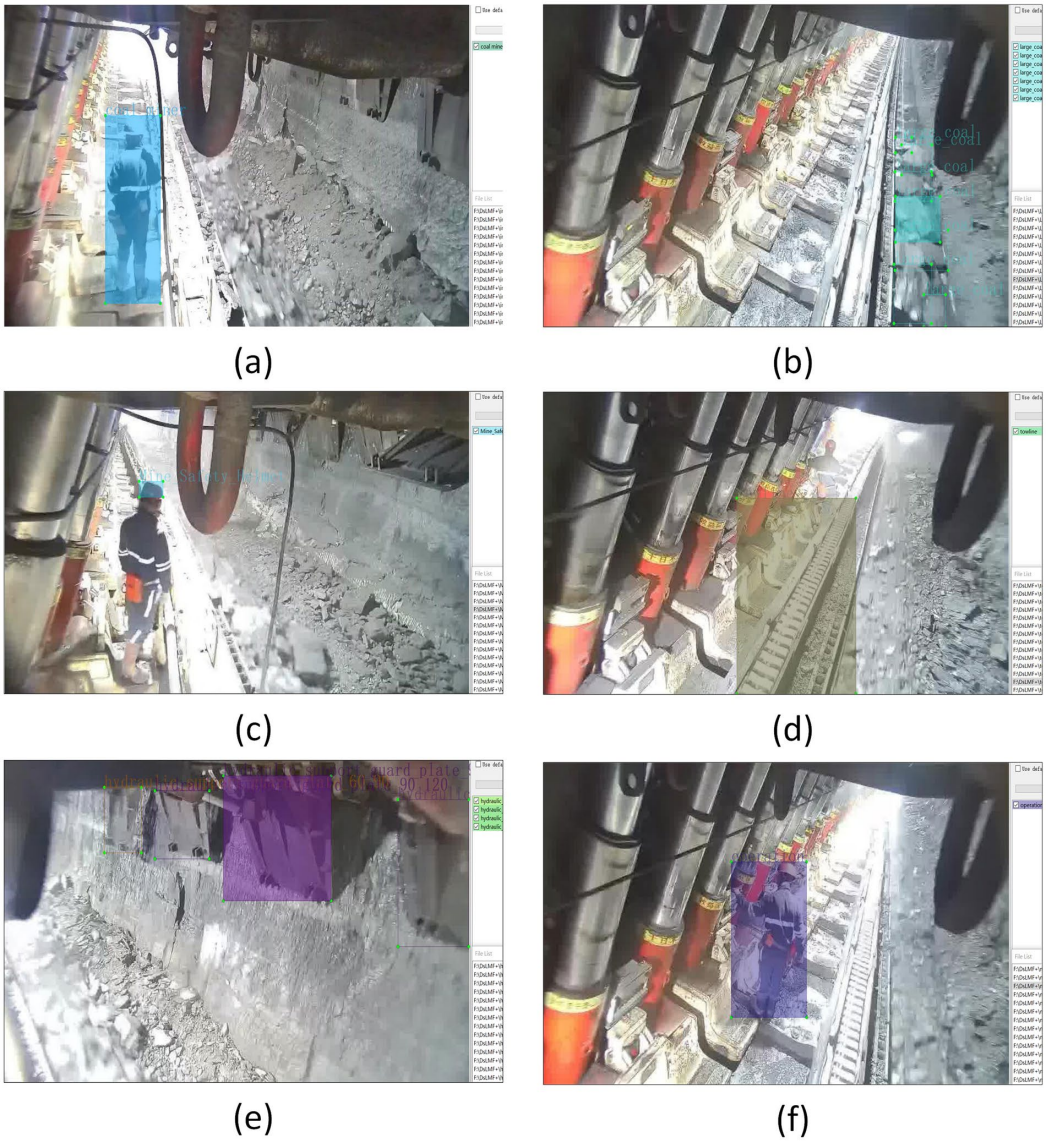
Label annotation for the dataset of fully mechanized longwall mining face: (**a**) Coal miner; (**b**) large coal; (**c**) mine safety helmet; (**d**) towline; (**e**) hydraulic support guard plate; (**f**) miners’ behaviors.

Finally, the filtered original image datasets were annotated using LabelImg software and named the label, and here we provide an official open source download link (https://github.com/heartexlabs/labelImg) for the Labelimg software. The researchers can set the label in YOLO, VOC or CreateML format and annotate the images according to the instructions provided by the official. In the process of labeling diverse kinds of datasets, the label order needed be determined accordingly. Once the label order is determined, the label order cannot be changed when open the software to label next time. If the order is changed, the label order of the dataset will be automatically changed to the current label order, and the original labeled annotations will appear in the current order, resulting in label confusion in the dataset. The LabelImg tool was used to annotate the training set and validation set in accordance with YOLO format, in the meanwhile, we also converted the YOLO datasets into COCO datasets through script files and retain. This work includes the datasets of the mine personnel, towline, mine safety helmet and large coal with the single-label annotation, as well as the hydraulic support guard plate and miners’ behaviors with multi-label annotations. Figure 2 shows the label annotations of coal miners, large coal, towline, mine safety helmet, miners’ behavior and supporting state of the hydraulic support guard plate.

**Fig. 3:**
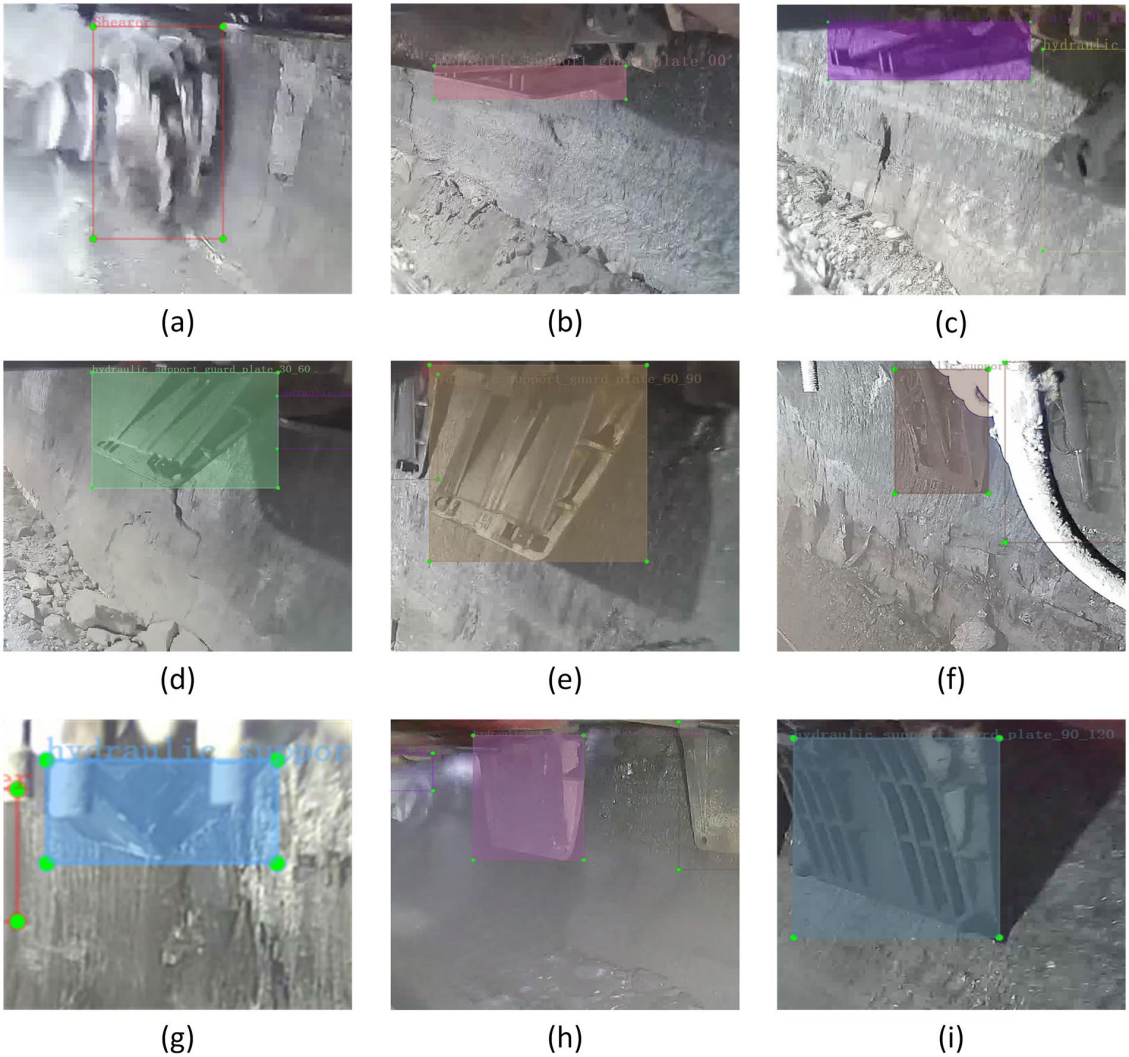
The dataset label annotations for the hydraulic support guard plate states. (**a**) Shearer; (**b**) hydraulic_support_guard_plate_00; (**c**) hydraulic_support_guard_plate_00_30; (**d**) hydraulic_support_guard_plate_30_60; (**e**) hydraulic_support_guard_plate_60_90; (**f**) hydraulic_support_guard_plate_90; (**g**) hydraulic_support_guard_plate_abnormal; (**h**) hydraulic_support_guard_plate_90_abnormal; (**i**) hydraulic_support_guard_plate_90_120.

The single-label datasets of the large coal, mine safety helmet, towline and mine personnel are named as large_coal, mine_safety_helmet, towline and coal miner, respectively. In order to judge whether there is movement interference between the shearer’s operation and the guard plate, the images are labeled according to the unfolding angle of the hydraulic support guard plate in this work, so as to obtain the support state information of the hydraulic support of the fully mechanized mining face. In the process of labeling the guard plate, the label types cover all angles of the hydraulic support guard plate. In order to ensure the accuracy of angle labeling, this work uses the built-in sensor in the hydraulic support of the fully mechanized mining face to detect and extract the angle information of the guard plate in real time. The extracted angle information is not only used to annotate the image of the guard plate in the dataset, but also to verify whether the annotated angle types of the guard plate are reasonable. Among which, In accordance with the different angle of unfolding of the hydraulic support guard plate, the supporting states of the hydraulic support guard plate are divided into eight kinds of type, which were respectively named as hydraulic_support_guard_plate_00, hydraulic_support_guard_plate_00_30, hydraulic_support_guard_plate_30_60, hydraulic_ support_guard_plate_60_90, hydraulic_support_guard_plate_90,hydraulic_support_guard_ plate_90_abnormal, hydraulic _support_guard_plate_90_120 and hydraulic_support_guard_ plate_abnormal. In order to judge whether there will be motion interference between the guard plate and the shearer, the label annotation for the image in which the shearer passing under the hydraulic support guard plate is also marked as Shearer. The involved dataset labels of the hydraulic support guard plate states are shown in Fig. 3.

Among them, hydraulic_support_guard_plate_00 state is the state when the guard plate is fully recovered and there is no interference with shearer operation. The numbers before and after the underline in hydraulic_support_guard_plate_00_30, hydraulic_support_guard_ plate_30_60 and hydraulic_support_guard_plate_60_90 respectively represent the angle range corresponding to the unfolding state of the guard plate. When the guard plate is in these three states, it will interfere with the shearer in operation. In hydraulic_support _guard_plate_90 state, when the unfolding angle corresponding to the state of the guard plate is 90°, the supporting plate is close to the coal wall, which can play a well supporting role on the coal wall and effectively prevent the occurrence of coal wall slab accident in the fully mechanized mining face. In hydraulic_support_guard_plate_abnormal state, there is a problem in the structure of the hydraulic support guard plate, which should be replaced in time. In hydraulic_support_guard_plate_90_abnormal state, the unfolding angle of the guard plate is 90°, and there is a small gap between the guard plate and the coal wall, so the support strength is not enough. In hydraulic_support_guard_plate_90_120 state, the unfolding angle of the guard plate is too large, which resulting in the gap between the guard plate and the coal wall is too large, and the support strength is not enough.

In order to ensure the universality and compatibility of this dataset, we collected the images of mine personnel, large coal, towline, mine safety helmet, miners’ behaviors and hydraulic support guard plate from multiple scenes, respectively. The image data of mine personnel came from 58 different scenes, the image data of large coal came from 18 different scenes, the image data of guard plate came from 159 different scenarios, the image data of towline images came from 65 different scenarios, the image data of mine safety helmet came from 85 different scenarios, and the image data of the miners’ behaviors came from 67 different scenarios. The DsLMF+ datasets built in this work are divided into training set and validation set at the ratio^32^ of 8:2. There are 30704 mine personnel images with 24563 images in training sets and 6141 in validation set, 21017 large coal images with 16813 images in training sets and 4204 in validation set, 21412 towline images with 17129 in training sets and 4283 in validation set, 20117 mine safety helmet images with 16093 in training set and 4024 in validation set, 24709 miners’ behavior images with 19767 in training sets and 4942 in validation set, and 20045 hydraulic support guard plates images with 16036 in training sets and 4009 in validation set. Tables 1–7 respectively describes the datasets of mine safety helmet, towline, coal miners, miners’ behavior, large coal and guard plate in multiple different scenarios.

**Table 1:** The summary of the training set and validation set for mine safety helmet.

Collection statistics	Characteristics	Training set						Validation set					
	Years	2020 to 2023						2020 to 2023					
	Data size(MB)	2247.41						570.07					
		Scenario	Count	Scenario	Count	Scenario	Count	Scenario	Count	Scenario	Count	Scenario	Count
Dataset label	Mine Safety Helmet	1	2783	30	26	59	90	1	716	30	6	59	18
		2	1099	31	29	60	2	2	252	31	8	60	2
		3	1636	32	36	61	28	3	398	32	7	61	9
		4	106	33	301	62	22	4	20	33	84	62	3
		5	1	34	8	63	5	5	0	34	3	63	3
		6	313	35	18	64	2	6	74	35	4	64	0
		7	55	36	115	65	29	7	9	36	25	65	5
		8	518	37	69	66	50	8	119	37	13	66	16
		9	229	38	14	67	88	9	50	38	5	67	25
		10	122	39	3	68	87	10	25	39	1	68	24
		11	68	40	10	69	19	11	14	40	2	69	5
		12	143	41	21	70	17	12	35	41	9	70	2
		13	240	42	5	71	1235	13	60	42	1	71	302
		14	224	43	27	72	7	14	56	43	3	72	0
		15	75	44	5	73	10	15	30	44	4	73	3
		16	893	45	59	74	211	16	42	45	14	74	59
		17	35	46	4	75	234	17	215	46	0	75	54
		18	54	47	5	76	169	18	11	47	0	76	51
		19	67	48	15	77	94	19	19	48	3	77	33
		20	44	49	83	78	4	20	8	49	21	78	1
		21	78	50	8	79	5	21	13	50	0	79	6
		22	102	51	7	80	38	22	18	51	0	80	12
		23	107	52	54	81	183	23	29	52	22	81	45
		24	49	53	899	82	295	24	17	53	232	82	75
		25	69	54	32	83	2	25	14	54	12	83	0
		26	241	55	207	84	1316	26	65	55	59	84	322
		27	171	56	49	85	11	27	40	56	11	85	0
		28	47	57	5			28	10	57	1		
		29	143	58	14			29	36	58	4		
		Total	16093					Total	4024

**Table 2:** The summary of the training set and validation set for towline.

Collection statistics	Characteristics	Training set						Validation set					
	Years	2020 to 2023						2020 to 2023					
	Data size (MB)	2495.77						624.4					
		Scenario	Count	Scenario	Count	Scenario	Count	Scenario	Count	Scenario	Count	Scenario	Count
Dataset label	towline	1	583	23	11	45	93	1	179	23	2	45	25
		2	1752	24	19	46	725	2	418	24	7	46	161
		3	923	25	11	47	12	3	193	25	4	47	4
		4	485	26	274	48	36	4	118	26	75	48	11
		5	663	27	17	49	98	5	168	27	3	49	35
		6	38	28	31	50	269	6	10	28	8	50	54
		7	47	29	8	51	1325	7	9	29	2	51	330
		8	34	30	111	52	80	8	8	30	22	52	10
		9	167	31	62	53	283	9	48	31	22	53	82
		10	209	32	118	54	86	10	43	32	33	54	20
		11	220	33	210	55	295	11	74	33	56	55	74
		12	154	34	145	56	103	12	48	34	40	56	19
		13	1112	35	929	57	288	13	275	35	243	57	74
		14	79	36	35	58	194	14	27	36	6	58	48
		15	31	37	153	59	36	15	8	37	26	59	10
		16	59	38	44	60	241	16	17	38	19	60	68
		17	667	39	18	61	20	17	176	39	3	61	4
		18	25	40	12	62	143	18	7	40	0	62	38
		19	9	41	16	63	181	19	3	41	5	63	38
		20	58	42	856	64	114	20	3	42	196	64	29
		21	14	43	17	65	47	21	12	43	6	65	9
		22	1625	44	409			22	402	44	116		
		Total	17129					Total	4283

**Table 3:** The summary of the training set and validation set for coal miner.

Collection statistics	Characteristics	Training set						Validation set					
	Years	2020 to 2023						2020 to 2023					
	Data size(MB)	3072						772					
		Scenario	Count	Scenario	Count	Scenario	Count	Scenario	Count	Scenario	Count	Scenario	Count
Dataset label	Coal miner	1	5067	21	95	41	23	1	1284	21	25	41	2
		2	1407	22	97	42	26	2	324	22	23	42	10
		3	2781	23	50	43	355	3	692	23	16	43	91
		4	1166	24	127	44	86	4	300	24	38	44	24
		5	2	25	43	45	2155	5	0	25	12	45	512
		6	93	26	30	46	151	6	28	26	4	46	38
		7	138	27	142	47	61	7	37	27	45	47	18
		8	140	28	19	48	474	8	39	28	2	48	131
		9	7	29	44	49	197	9	3	29	12	49	47
		10	28	30	232	50	35	10	4	30	51	50	8
		11	4	31	244	51	1228	11	0	31	62	51	293
		12	52	32	330	52	23	12	13	32	78	52	3
		13	409	33	2068	53	693	13	107	33	521	53	193
		14	184	34	98	54	105	14	36	34	18	54	27
		15	7	35	22	55	469	15	4	35	2	55	117
		16	98	36	43	56	947	16	16	36	14	56	251
		17	14	37	84	57	7	17	9	37	24	57	1
		18	152	38	17	58	1849	18	21	38	6	58	457
		19	32	39	21			19	11	39	9		
		20	33	40	59			20	9	40	19		
		Total	24563					Total	6141

**Table 4:** The summary for the datasets of miners’ behavior.

Collection statistics	Characteristics					Training set				Validation set			
	Years					2020 to 2023				2020 to 2023			
	Data size (MB)					3983.68				1009.43			
		Scenario	label type	Scenario	label type	Scenario	count	Scenario	Count	Scenario	Count	Scenario	Count
Dataset label	miners’ behavior	1	ABCDEFH	35	ABCEFH	1	1324	35	354	1	347	35	81
		2	ABCDEF	36	ACF	2	1233	36	64	2	310	36	19
		3	ACDEF	37	AC	3	174	37	5	3	56	37	1
		4	ABCDEFH	38	ACEF	4	1050	38	89	4	248	38	20
		5	ABCEF	39	ACF	5	482	39	18	5	108	39	3
		6	ACDF	40	A	6	126	40	3	6	25	40	0
		7	C	41	ABCE	7	3	41	57	7	0	41	22
		8	ABCDEF	42	AC	8	33	42	63	8	12	42	12
		9	ABCDEF	43	ACF	9	420	43	76	9	100	43	22
		10	ACDEF	44	ABCEF	10	164	44	633	10	37	44	151
		11	ACEF	45	AC	11	41	45	35	11	10	45	10
		12	ACDF	46	ACEFH	12	67	46	56	12	12	46	19
		13	ACD	47	ACEF	13	36	47	107	13	8	47	16
		14	B	48	A	14	16	48	14	14	1	48	8
		15	A	49	ABCEFH	15	3	49	4678	15	1	49	1200
		16	AC	50	C	16	24	50	2	16	9	50	0
		17	ACEF	51	AC	17	89	51	35	17	23	51	11
		18	ACEFH	52	ABCF	18	630	52	355	18	158	52	91
		19	ACDF	53	ABCEF	19	241	53	390	19	64	53	95
		20	ACF	54	ABCEF	20	84	54	49	20	18	54	20
		21	A	55	ACEF	21	5	55	449	21	1	55	83
		22	CE	56	ABCEF	22	66	56	166	22	17	56	82
		23	AC	57	ACEF	23	10	57	69	23	3	57	14
		24	AE	58	ACEFH	24	2	58	217	24	0	58	46
		25	A	59	ABCEF	25	17	59	926	25	6	59	231
		26	A	60	ABCDEFH	26	10	60	1104	26	5	60	298
		27	A	61	A	27	9	61	7	27	3	61	1
		28	CDEH	62	ABCEFH	28	4	62	1156	28	2	62	254
		29	CEF	63	ACEF	29	10	63	26	29	2	63	8
		30	AF	64	G	30	13	64	169	30	3	64	36
		31	AC	65	G	31	9	65	63	31	4	65	20
		32	A	66	G	32	20	66	6	32	2	66	1
		33	ABCEFH	67	G	33	1818	67	45	33	447	67	10
		34	CF			34	48			34	15		
						Total	19767			Total	4942		

Note that in order to facilitate statistics, the different label annotations are marked for the different behaviors of coal miners. Among which, A,walking; B,sitting; C,standing; D,operation; E,stoop; F,lean against; G,tumble; H,climb over.

**Table 5:** The summary of the training set and validation set for large coal.

Collection statistics	Characteristics	Training set						Validation set					
	Years	2020 to 2023						2020 to 2023					
	Data size (MB)	751.5						187					
		Scenario	Count	Scenario	Count	Scenario	Count	Scenario	Count	Scenario	Count	Scenario	Count
Dataset label	Large Coal	1	448	7	62	13	84	1	134	7	13	13	22
		2	6965	8	1018	14	1628	2	1718	8	243	14	437
		3	1131	9	297	15	1236	3	296	9	74	15	309
		4	415	10	521	16	47	4	117	10	127	16	7
		5	89	11	1331	17	1324	5	9	11	301	17	345
		6	141	12	36	18	40	6	36	12	7	18	9
		Total	16813					Total	4204

**Table 6:** The summary for the datasets of hydraulic support guard plate that from scenario 1 to scenario 108.

Collection statistics	Characteristics							Training set						Validation set					
	Years							2020 to 2023						2020 to 2023					
	Data size (MB)							1434.19						360.14					
		Scenario	label type	Scenario	label type	Scenario	label type	Scenario	Count	Scenario	Count	Scenario	Count	Scenario	Count	Scenario	Count	Scenario	Count
Dataset label	Hydraulic support guard plate	1	ABCDEF	37	ACDEF	73	ACD	1	47	37	43	73	19	1	19	37	6	73	3
		2	ABCDEF	38	ABCDEF	74	BCDEFI	2	130	38	63	74	38	2	51	38	12	74	9
		3	CDE	39	BCDEF	75	CDEF	3	27	39	27	75	31	3	5	39	13	75	8
		4	CDEF	40	ABCDEF	76	BCDEF	4	13	40	63	76	25	4	4	40	23	76	5
		5	ABCDE	41	BCDEF	77	CDEF	5	21	41	39	77	26	5	6	41	5	77	8
		6	AH	42	ABCDE	78	CDI	6	18	42	47	78	7	6	4	42	16	78	1
		7	BCDE	43	ABCDEF	79	ABCDEF	7	44	43	54	79	28	7	18	43	9	79	7
		8	ABCDEF	44	ABCDEF	80	BCDEF	8	115	44	53	80	23	8	16	44	9	80	4
		9	ABCDEF	45	ABCDEF	81	ABCDEF	9	74	45	71	81	84	9	14	45	22	81	34
		10	ABCDEFG	46	ABCDEF	82	F	10	89	46	52	82	6	10	31	46	9	82	4
		11	ABCDE	47	BCDE	83	CDE	11	27	47	39	83	55	11	12	47	14	83	7
		12	ABCDE	48	ACEH	84	ABCDEF	12	36	48	182	84	55	12	11	48	42	84	7
		13	ABCDEF	49	AEH	85	ABCDE	13	58	49	149	85	7	13	16	49	51	85	3
		14	BCDEF	50	ABCDEFGH	86	ABCDE	14	26	50	818	86	14	14	11	50	197	86	6
		15	ABCDEF	51	ABCDEFI	87	BCD	15	88	51	709	87	20	15	33	51	173	87	11
		16	ABCDE	52	ABCDEFHI	88	DEF	16	24	52	472	88	241	16	5	52	106	88	69
		17	BCDEF	53	ABCFH	89	DEF	17	41	53	256	89	383	17	4	53	43	89	85
		18	ABCDEF	54	F	90	DEF	18	29	54	51	90	151	18	9	54	13	90	57
		19	ABCDEF	55	AC	91	CF	19	84	55	23	91	33	19	17	55	6	91	5
		20	ABCDEF	56	ABEFH	92	BDEHI	20	14	56	147	92	429	20	2	56	29	92	105
		21	ABCDE	57	BH	93	BCDH	21	23	57	304	93	311	21	7	57	73	93	60
		22	ABCDEF	58	CDI	94	BCDEHI	22	97	58	57	94	419	22	23	58	23	94	98
		23	ABCDEF	59	ABCDEFH	95	CDEI	23	60	59	108	95	258	23	8	59	21	95	71
		24	ABCDEF	60	CDFH	96	BCDEHI	24	76	60	108	96	517	24	14	60	32	96	131
		25	BCDEF	61	ABCDEFHI	97	D	25	36	61	461	97	5	25	8	61	116	97	0
		26	BDEFGH	62	ABCDEH	98	DEF	26	72	62	53	98	52	26	17	62	23	98	5
		27	ABCDEF	63	ACDE	99	DEF	27	28	63	99	99	103	27	6	63	25	99	20
		28	EF	64	ABCDEH	100	E	28	11	64	106	100	14	28	1	64	29	100	4
		29	ABCDEF	65	ABCDF	101	DE	29	44	65	77	101	131	29	15	65	17	101	28
		30	BCDE	66	BCDE	102	DEF	30	32	66	95	102	68	30	8	66	29	102	14
		31	CDEF	67	BCDFI	103	DE	31	11	67	21	103	48	31	2	67	9	103	13
		32	EF	68	CDFI	104	ABCDEFH	32	7	68	27	104	330	32	1	68	3	104	84
		33	ABCDEF	69	EF	105	F	33	25	69	38	105	40	33	3	69	6	105	17
		34	ABCDE	70	ABCDEF	106	ABCEF	34	13	70	46	106	53	34	4	70	9	106	11
		35	ABCDEF	71	BDEF	107	ABCD	35	173	71	144	107	198	35	48	71	35	107	40
		36	BCDEF	72	DEF	108	DEF	36	40	72	4	108	222	36	8	72	2	108	64
								Total	11303	Total	2809								

Note that in order to facilitate statistics, the different label annotations are marked for the different closing states of the guard plate. Among which, A represents hydraulic_support_guard_plate_00, B represents hydraulic_support_guard_plate_00_30, C represents hydraulic_support_guard_ plate_30_60, D hydraulic_support_guard_plate_60_90, E represents hydraulic_support_guard_plate_90, F represents hydraulic_support_guard_ plate_90_120, G represents hydraulic_support_guard_plate_abnormal, H represents Shearer and I represents hydraulic_support_ guard_plate_90_abnormal.

**Table 7:** The summary for the datasets of hydraulic support guard plate that from scenario 109 to scenario 159.

Collection statistics	Characteristics							Training set						Validation set					
	Years							2020 to 2023						2020 to 2023					
	Data size (MB)							1434.19						360.14					
		Scenario	label type	Scenario	label type	Scenario	label type	Scenario	Count	Scenario	Count	Scenario	Count	Scenario	Count	Scenario	Count	Scenario	Count
Dataset label	Hydraulic support guard plate	109	DE	126	DF	143	CDE	109	45	126	78	143	14	109	10	126	12	143	10
		110	BCDEFI	127	DF	144	B	110	854	127	32	144	14	110	213	127	9	144	6
		111	DE	128	DE	145	CB	111	38	128	74	145	16	111	8	128	11	145	4
		112	BCDEFI	129	CDEH	146	C	112	269	129	126	146	16	112	82	129	41	146	2
		113	BCDEH	130	D	147	ABCH	113	44	130	54	147	142	113	9	130	19	147	33
		114	DEFI	131	D	148	ABCDFH	114	11	131	47	148	178	114	2	131	8	148	43
		115	BCDH	132	BCDEH	149	ABCH	115	46	132	20	149	214	115	12	132	7	149	65
		116	BCDEFI	133	DE	150	ABCD	116	244	133	51	150	148	116	57	133	8	150	45
		117	B	134	ABH	151	ABCH	117	60	134	82	151	48	117	9	134	22	151	9
		118	BE	135	AH	152	ABC	118	67	135	147	152	39	118	22	135	34	152	10
		119	B	136	ABDEH	153	ABCDH	119	42	136	18	153	113	119	16	136	5	153	28
		120	BCDE	137	B	154	ABCDEF	120	25	137	5	154	175	120	6	137	0	154	41
		121	BH	138	CE	155	AB	121	12	138	13	155	49	121	3	138	2	155	13
		122	CDEF	139	CE	156	AB	122	366	139	31	156	4	122	97	139	7	156	5
		123	DE	140	ABCDEH	157	BDF	123	86	140	165	157	10	123	20	140	38	157	1
		124	E	141	ACD	158	DE	124	103	141	38	158	2	124	25	141	5	158	1
		125	CDEHI	142	CDE	159	BD	125	215	142	35	159	8	125	54	142	7	159	4
								Total	4733					Total	1200				

Note that in order to facilitate statistics, the different label annotations are marked for the different closing states of the guard plate. Among which, A represents hydraulic_support_guard_plate_00, B represents hydraulic_support_guard_plate_00_30, C represents hydraulic_support_guard_plate_30_60, D hydraulic_support_guard_plate_60_90, E represents hydraulic_support_guard_plate_90, F represents hydraulic_support_guard_ plate_90_120, G represents hydraulic_support_guard_plate_abnormal, H represents Shearer and I represents hydraulic_support_guard_plate_90_abnormal.

## Data Records

The DsLMF+ dataset of the coal mine image in the fully mechanized longwall mining face has been publicly available at the figshare data repository^33^. Data annotations include YOLO format and COCO format. Among them, the image and label files of the dataset in YOLO format are stored as follows: the folder names of each dataset in data2023_yolo are respectively coal_miner_data2023_yolo, large_coal_data2023_yolo, mine_safety_helmet_data2023_yolo, towline_data2023_yolo,miner_behavior_data2023_yolo and hydraulic_support_guard_plate _data2023_yolo. Each folder contains the picture folders and label folders that named as images and labels, in which respectively stores image data and label data. These folders also contain training set folders and verification set folders. The information contained in the label data mainly includes data type, number of labels and label coordinates.

The image and label files of the dataset in COCO format are stored as follows: the folder names of each dataset in data2023_coco are respectively coal_miner_data2023_coco, large_coal_data2023_coco, mine_safety_helmet_data2023_coco, towline_data2023_coco, miner_behavior_data2023_coco and hydraulic_support_guard_plate_data2023_coco. Each of these folders contains the training set image folder, verification set image folder and label folder respectively named as train2017, val2017 and annotations, which are used to store training set pictures, verification set pictures and label files. The information contained in COCO label files contains file name, image width and height, label category and label coordinates, etc.

In addition, the files coal_miner_DsLMF, large_coal_DsLMF, mine_safety_helmet_DsLMF, towine_DsLMF, miner_behavior_DsLMF and hydraulic_support_guard_plate_DsLMF are provided to be used to better distinguish the images of mine personnel, large coal, towline, miners’ behavior, mine safety helmet and guard plate in different scenarios in DsLMF+ datasets, and the image index corresponding to the different scenes are given in the files.

## Technical Validation

To ensure the reliability of the DsLMF+ dataset in this work, we also conducted a comprehensive manual review of all images and their corresponding label annotation. The specific review method is as follows: five members with rich working experience in the coal mining field are selected to check the image dataset and label information one by one to see whether there are missing or wrong labels. At the same time, in order to ensure the quality and application effect of the dataset, the five members uniformly reviewed the controversial images in the dataset, such as the size threshold of large coal, the angle involved in the guard plate image and its label, the label veracity of the downhole towline, coal personnel behaviour and the mine safety helmet. Through the collective voting of the five members, the review work of the dataset was completed.

DsLMF+ dataset have provided two types of datasets formats of YOLO and COCO, which make it convenient to be applied for the currently popular top-ranked target detection neural networks. In order to verify the feasibility of the constructed dataset, this work selected YOLOv7^34^, DETA^35^ and ViT-Adapter-L^36^ three top deep learning network from the COCO target detection ranking list, and conducted model training and verification on the DsLMF+ dataset. The access links of DETA, ViT-Adapter-L and YOLOv7 that used to verify the datasets are respectively https://github.com/jozhang97/deta, https://github.com/czczup/vit-adapter and https://github.com/wongkinyiu/yolov7. The DsLMF+ datasets were trained on a machine with Intel(R) Xeon(R) Gold 6330 CPU, RTX A5000 GPU and Ubantu18.04. The hyper-parameters of the above three target detection algorithms were on the reference to the recommended default values. To suit the dataset, some hyper-parameter values such as width, height, batch size, initial learning rate and Epochs are modified. This change was implemented in accordance to the recommendations from the initial YOLOv7, DETA, and ViT-Adapter-L research.

**Fig. 4:**
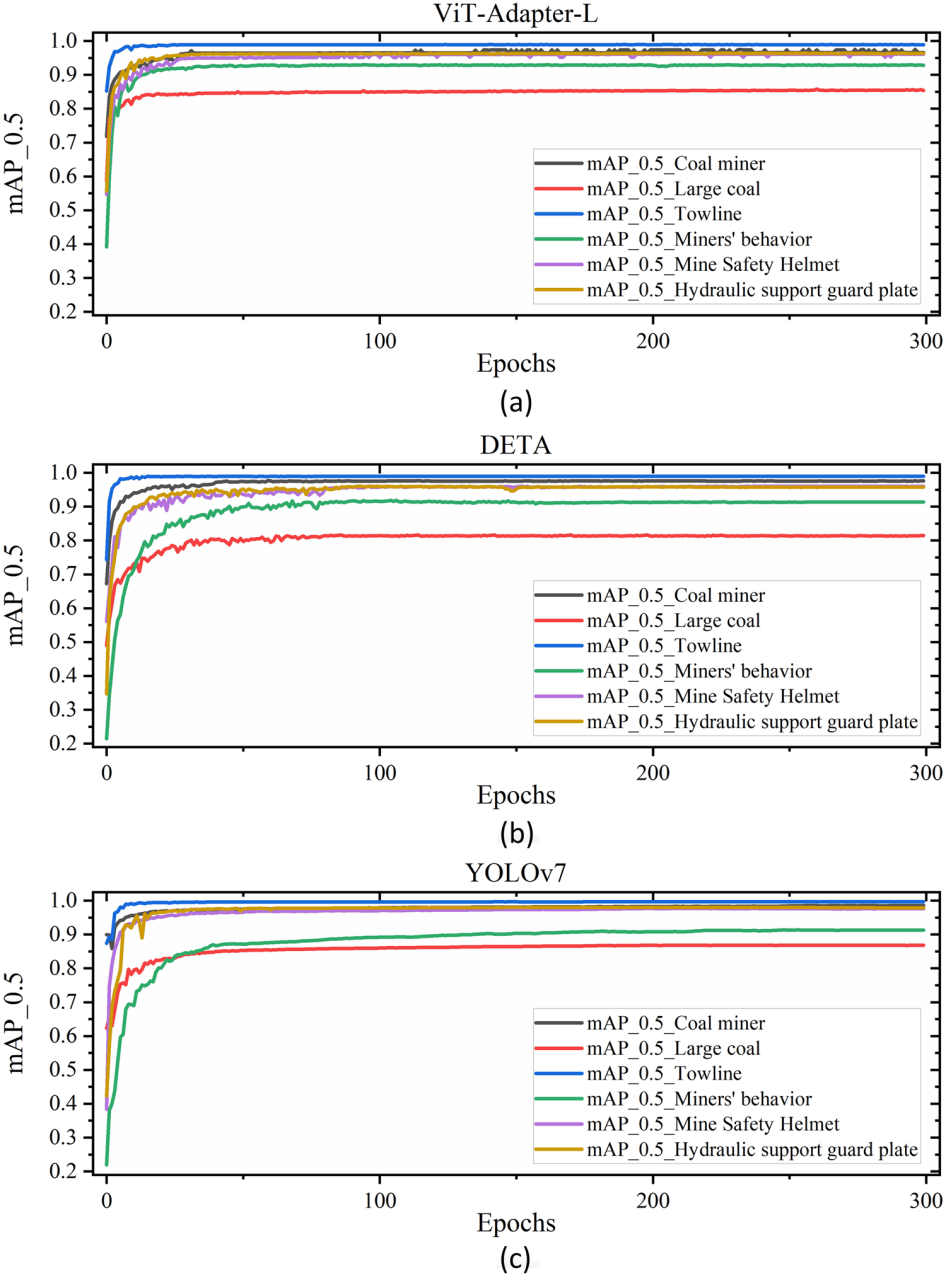
The validation mAp value curve of the models ViT-Adapter-L, DETA and YOLOv7 on the coal miners, large coal, towline, mine safety helmet, hydraulic support guard plate and miners’ behaviours in the.

**Fig. 5:**
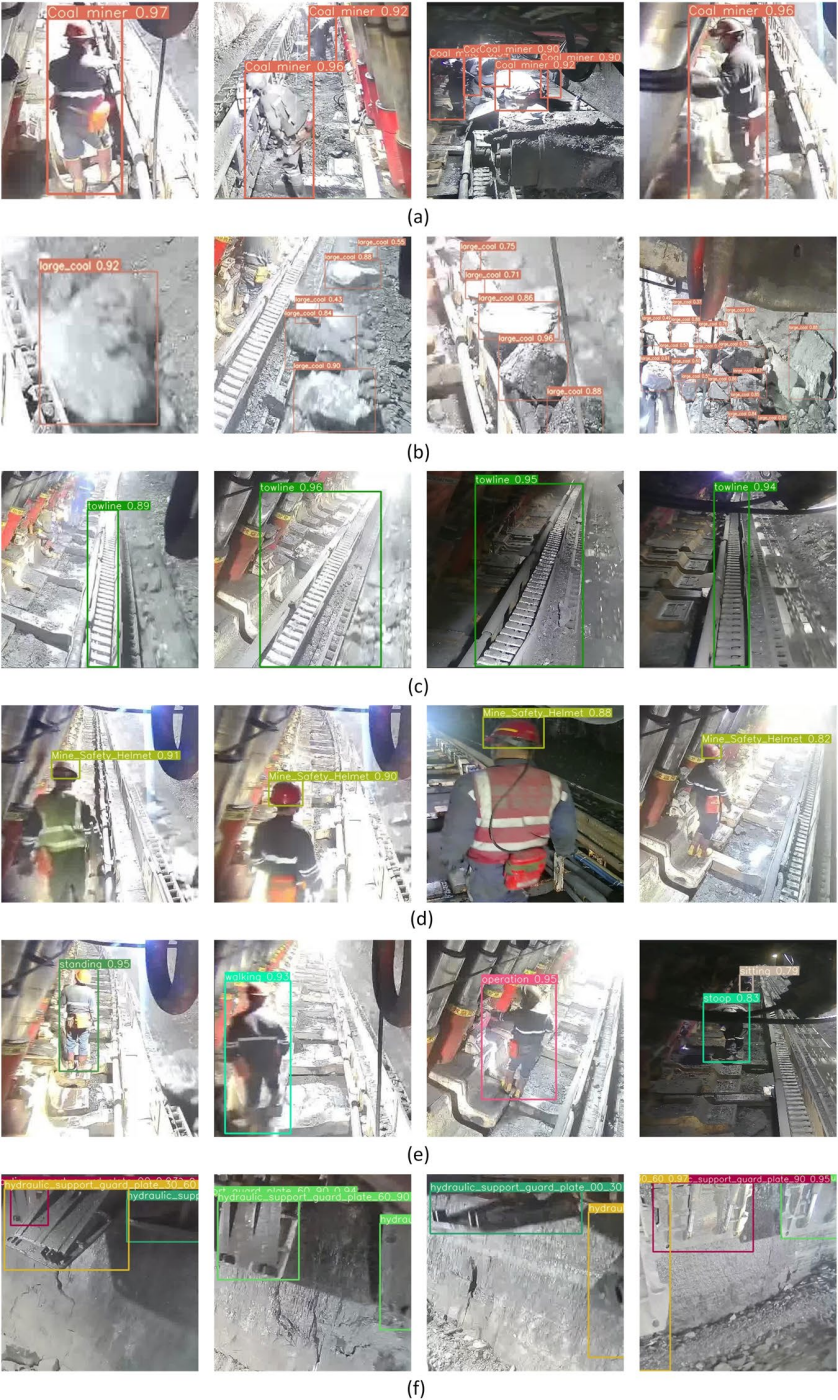
The validation of the DsLMF+ datasets by using of the models ViT-Adapter-L, DETA and YOLOv7. (**a**) coal miners; (**b**) large coal;(**c**) towline; (**d**) mine safety helmet; (**e**) miners’ behaviours; (**f**) hydraulic support guard plate.

For the dataset verification, the coal miners, large coal, towline, mine safety helmet, hydraulic support guard plate and miners’ behaviours in the datasets are trained and evaluated. The image height and width of the input image are both resized to 640 in the network training. Table 8 presents the benchmark result of ViT-Adapter-L, DETA and YOLOv7 on the DsLMF+ datasets. Figure 4 shows the graphs of the three model’s performance during validation, the mAP value curves of each target detection network model. The mAP values of YOLOv7 detection model can respectively reach 0.986, 0.976, 0.978, 0.868, 0.913 and 0.997, the mAP values of DETA detection model can respectively reach 0.976, 0.960, 0.958, 0.815, 0.914 and 0.989, and the mAP values of ViT-Adapter-L detection model can respectively reach 0.966, 0.961, 0.963, 0.854, 0.928 and 0.989. The above mAP values indicate that the models have good performance, and the DsLMF+ dataset performs well under YOLOv7, DETA and ViT-Adapter-L. The deployed YOLOv7, DETA and ViT-Adapter-L have been respectively used to randomly extract and detect the 6 categories of images of coal miners, large coal, towline, mine safety helmet, hydraulic support guard plate and miners’ behaviours in the DsLMF+ dataset, and the identified target detection results are shown in Fig. 5, the detection effect and accuracy demonstrated the reliability and practicability of DsLMF+ datasets.

**Table 8:** Benchmark of ViT-Adapter-L, DETA and YOLOv7 performed on coal miners, large coal, towline, mine safety helmet, hydraulic support guard plate and miners’ behaviours in the datasets DsLMF+.

Category of DsLMF+	ViT-Adapter-L				DETA				YOLOv7			
	AP0.5	AP0.75	AP0.5:0.95	AR0.5:0.95	AP0.5	AP0.75	AP0.5:0.95	AR0.5:0.95	P	R	mAP0.5	mAP0.5:0.95
Coal miners	0.966	0.808	0.702	0.739	0.976	0.787	0.684	0.781	0.965	0.968	0.986	0.773
Mine safety helmet	0.961	0.722	0.624	0.673	0.960	0.667	0.601	0.707	0.942	0.958	0.976	0.679
Hydraulic support guard plate	0.963	0.853	0.753	0.807	0.958	0.839	0.723	0.811	0.972	0.927	0.978	0.813
Large Coal	0.854	0.591	0.532	0.648	0.815	0.548	0.500	0.652	0.814	0.776	0.868	0.572
Miners’ behaviours	0.928	0.858	0.714	0.794	0.914	0.862	0.718	0.824	0.880	0.880	0.913	0.752
Towline	0.989	0.968	0.871	0.898	0.989	0.963	0.816	0.866	0.995	0.997	0.997	0.916

Moreover, we will further expand the DsLMF+ dataset to make the dataset have better applicability and universality in the fully mechanized coal mining face. We also encourage other researchers in coal mine field to expand and improve the DsLMF+ dataset. The coal mine image dataset produced in this work is of great significance for the application of deep learning object detection algorithm for the intelligent identification and classification of abnormal conditions for underground mining, which aims to support further research and advancement of intelligence in the fully mechanized longwall mining face .

**Table 9:** The overview of the site-packages for ViT-Adapter-L, DETA and YOLOv7.

DETA		ViT-Adapter-L		YOLOv7	
site-packages	versions	site-packages	versions	site-packages	versions
Python	3.8	Python	3.8	Python	3.8
Pytorch	1.9.0	Pytorch	1.9.0	Pytorch	1.11.0
Torchvision	0.10.0	Torchvision	0.10.0	Torchvision	0.12.0
Cudatoolkit	11.1.0	Cudatoolkit	11.1.0	Cudatoolkit	11.3.0
Timm	0.6.12	mmcv-full	1.4.2	matplotlib	3.5.2
Scipy	1.9.3	Timm	0.4.12	Pillow	9.1.1
Pycocotools	2.0.6	mmdet	2.22.0	opencv-python	4.6.0.66
Pillow	8.3.2	opencv-python	4.6.0.66	scipy	1.8.1
Tqdm	4.61.2	Pillow	8.3.2	protobuf	3.19.4

## Data Availability

DsLMF+ datasets are publicly available at the figshare data repository^33^, and the code for automatically filtering is also published alongside the dataset, archived as “DsLMF.7z”. Furthermore, the annotation tool Labelimg can be accessed and downloaded through the official website link https://github.com/heartexlabs/labelImg, the specific usage can refer to the corresponding README file. The codes used for training and validation of the DsLMF+ datasets in this work adopts DETA, ViT-Adapter-L and YOLOv7 official published open source scripts, and the code of the above three deep learning network for dataset verification can be accessed via the following website link (https://github.com/jozhang97/deta), (https://github.com/czczup/vit-adapter), and (https://github.com/wongkinyiu/yolov7). Table 9 presents the required site-packages and their corresponding versions for the above three different networks. The software packages can be downloaded according to README files under the corresponding links on different networks, and can be installed with the python package installer (pip). Researchers can complete the label format conversion from YOLO format to COCO format, by visiting the following link (https://github.com/RapidAI/YOLO2COCO), the link provides the label format conversion code and the README file that can be used as a reference.
