# Publisher Correction: An open dataset for intelligent recognition and classification of abnormal condition in longwall mining

**DOI:** 10.1038/s41597-024-03713-2

**Published:** 2024-08-06

**Authors:** Wenjuan Yang, Xuhui Zhang, Bing Ma, Yanqun Wang, Yujia Wu, Jianxing Yan, Yongwei Liu, Chao Zhang, Jicheng Wan, Yue Wang, Mengyao Huang, Yuyang Li, Dian Zhao

**Affiliations:** 1 School of Mechanical Engineering, Xi’an University of Science and Technology, No.58, Mid-Yanta Road, Xi’an, 710054 China; 2 Shaanxi Key Laboratory of Mine electromechanical equipment intelligent Detection and control, No.58, Yanta Road, Xi’an, 710054 China; 3 MARCO automatic control system development Co., LTD, No.20, Fenghui South Road, Xi’an, 710054 China

Correction to: *Scientific Data* 10.1038/s41597-023-02322-9, published online 27 June 2023

In this article the link provided for ref. 33 was incorrect and should have been ‘10.6084/m9.figshare.c.6307599.v1’. The original article has been corrected.

